# Lightweight and Real-Time Driver Fatigue Detection Based on MG-YOLOv8 with Facial Multi-Feature Fusion

**DOI:** 10.3390/jimaging11110385

**Published:** 2025-11-01

**Authors:** Chengming Chen, Xinyue Liu, Meng Zhou, Zhijian Li, Zhanqi Du, Yandan Lin

**Affiliations:** 1 College of Engineering Science and Technology, Shanghai Ocean University, Shanghai 201306, China; m230851608@st.shou.edu.cn (X.L.); m220851466@st.shou.edu.cn (M.Z.); zjli@shou.edu.cn (Z.L.); zqdu@shou.edu.cn (Z.D.); 2 Department of Illuminating Engineering & Light Sources, School of Information Science and Technology, Fudan University, Shanghai 201203, China; ydlin@fudan.edu.cn

**Keywords:** fatigue driving, YOLOv8, face detection, multi-feature fusion

## Abstract

Driver fatigue is a primary factor in traffic accidents and poses a serious threat to road safety. To address this issue, this paper proposes a multi-feature fusion fatigue detection method based on an improved YOLOv8 model. First, the method uses an enhanced YOLOv8 model to achieve high-precision face detection. Then, it crops the detected face regions. Next, the lightweight PFLD (Practical Facial Landmark Detector) model performs keypoint detection on the cropped images, extracting 68 facial feature points and calculating key indicators related to fatigue status. These indicators include the eye aspect ratio (EAR), eyelid closure percentage (PERCLOS), mouth aspect ratio (MAR), and head posture ratio (HPR). To mitigate the impact of individual differences on detection accuracy, the paper introduces a novel sliding window model that combines a dynamic threshold adjustment strategy with an exponential weighted moving average (EWMA) algorithm. Based on this framework, blink frequency (BF), yawn frequency (YF), and nod frequency (NF) are calculated to extract time-series behavioral features related to fatigue. Finally, the driver’s fatigue state is determined using a comprehensive fatigue assessment algorithm. Experimental results on the WIDER FACE and YAWDD datasets demonstrate this method’s significant advantages in improving detection accuracy and computational efficiency. By striking a better balance between real-time performance and accuracy, the proposed method shows promise for real-world driving applications.

## 1. Introduction

Fatigue driving refers to the impairment of a driver’s physiological and psychological functions due to sleep deprivation or prolonged driving, leading to slower reaction times and diminished driving skills. This increases the risk of delayed operations and imprecise steering corrections, significantly increasing the risk of traffic accidents [1,2,3]. To improve road safety, researchers worldwide are actively developing real-time driver state monitoring systems to detect fatigue status and issue timely warnings.

Extensive research has been carried out both domestically and internationally in the field of driver fatigue detection, yielding significant advancements and valuable insights. The primary methods for fatigue driving detection can currently be categorized into three types: (1) methods based on driver facial features [4,5], (2) methods based on driver physiological features [6,7], and (3) methods based on vehicle driving information [8,9]. As computer vision, artificial intelligence, and CPU/GPU hardware technology continue to develop, fatigue detection methods based on facial features have made significant progress, achieving high accuracy and real-time performance under ideal conditions. Under fatigued conditions, a driver’s facial features typically undergo noticeable changes, such as increased blink frequency, reduced eye opening, and frequent yawning [10,11]. This method uses machine vision technology and convolutional neural networks to extract features and assess fatigue from driver images. It offers advantages such as non-invasiveness, efficiency, and low cost. However, in complex real-world driving scenarios, the accuracy of these methods is still challenged by factors such as individual differences, lighting changes, and occlusions. Further research is necessary to enhance their robustness and reliability in actual application environments.

Current fatigue detection methods that are based on facial features, such as the eyes, mouth, and head posture, primarily rely on extracting various features and comparing them to fixed thresholds that are set for each feature. However, since a uniform average threshold is typically used for each feature, this approach struggles to adapt to individual differences among drivers, thereby affecting the overall accuracy of detection. Additionally, if fatigue assessment relies solely on one type of feature (e.g., the eyes or mouth), the method is not adaptable in complex driving scenarios and is easily influenced by environmental factors, such as pose occlusion and changes in lighting conditions. Furthermore, achieving a balance between high detection accuracy and real-time performance in limited computational environments remains a critical challenge for fatigue detection systems when deployed in real-world embedded settings. System design must focus not only on improving algorithm performance, but also on prioritizing model lightweighting and response efficiency to meet the real-time operational requirements of embedded platforms.

To address the aforementioned challenges, this paper proposes a face-based, multi-feature driver-fatigue detection algorithm built upon an improved YOLOv8 architecture. Our principal contributions are as follows:

- The improved YOLOv8 model introduces the GELAN module, the Mixed Local Channel Attention (MLCA) mechanism, and the EIoU loss function. These enhancements improve face detection accuracy in complex scenes and effectively optimize network structure and inference efficiency.
- A fatigue detection metric system that integrates multiple dynamic facial features has been developed. This system comprehensively incorporates blink frequency (BF), yawn frequency (YF), nod frequency (NF), and eyelid closure percentage (PERCLOS). This replaces traditional, single-metric discrimination methods and significantly improves the stability and adaptability of the fatigue detection system under multiple scenarios and subjects.
- A novel sliding window model based on a dynamic threshold adjustment strategy and an exponential weighted moving average (EWMA) algorithm has been proposed. This model can better adapt to physiological differences among drivers and the temporal evolution patterns of fatigue behavior. Thus, it enhances the stability and individual adaptability of fatigue detection.
- Systematic experimental validation was conducted using the publicly available YAWDD fatigue driving dataset and a simulated driving platform. The results demonstrate that the proposed method exhibits excellent detection accuracy and real-time processing capabilities across various driving environments, showcasing its strong potential for practical application.

## 2. Related Work

In recent years, with the rapid advancement of deep learning and computer vision, driver fatigue detection has evolved from traditional static image analysis toward dynamic behavior recognition and multimodal feature fusion. Research in this domain primarily focuses on two core aspects: effective feature representation and optimization of detection performance. In the broader field of general computer vision, multi-feature fusion techniques have achieved remarkable progress, providing essential methodological insights for fatigue detection. For instance, [12] proposed a unified framework for video fusion that enhances temporal feature modeling through multi-frame learning and large-scale benchmarking; study [13] explored the integration of vision–language models for image fusion, strengthening cross-modal semantic alignment; [14] introduced an equivariant multimodality image fusion approach that improves robustness to pose and illumination variations through geometric invariance design; reference [15] employed denoising diffusion models to achieve consistent multi-modality fusion under complex noise conditions; and study [16] developed a correlation-driven dual-branch feature decomposition mechanism that refines fused representations by separating shared and modality-specific features. Although these studies were not explicitly designed for driver fatigue detection, they provide a solid theoretical foundation and valuable technical references for developing multi-feature fusion strategies in this domain. Building upon these advances, current research on driver fatigue detection increasingly emphasizes the synergistic utilization of multimodal features to achieve more robust and generalizable recognition of fatigue-related behaviors.

Early studies primarily inferred fatigue from static spatial cues, such as the opening and closing of the eyes and mouth. Viola et al. [17] introduced the AdaBoost cascade classifier to achieve efficient and rapid face detection. With the advent of convolutional neural networks and contemporary object detectors, models such as including Faster R-CNN [18], SSD [19], improved MTCNN [20], and YOLO [21] have gained widespread adoption. Wang et al. [22] combined spatial eye-state cues with multi-feature temporal fusion for fatigue recognition, while Zhang et al. [23] used MTCNN to localise facial landmarks. Ji et al. [11] designed ESR and MSR networks on top of MTCNN to identify spatial differences in facial states, and Adhinata et al. [24] paired FaceNet embeddings with a multi-class SVM to analyse temporal blinking patterns. Meanwhile, Deng et al. [25] employed RetinaFace to enhance spatial detection precision, and Liu et al. [26] fused MB-LBP texture features with AdaBoost for graded detection. Despite these advances, many approaches remain sensitive to illumination and occlusion, and do not sufficiently exploit joint spatiotemporal cues. In parallel, the YOLO family has catalysed real-time detection. Li et al. [27] combined YOLOv3, Dlib and SVM, validating the real-time capabilities on DSD and WIDER FACE. You et al. [28] applied this framework to YawDD, further enhancing spatiotemporal matching, underscoring YOLO’s advantages in coordinated spatiotemporal modeling and lightweight, real-time detection.

In existing studies utilizing driver behavior data, spatial and temporal features remain the dominant focus. Researchers generally acknowledge that spatial features extracted from single-frame images are insufficient to capture the dynamic evolution of fatigue behaviors, necessitating the integration of temporal information for precise recognition. For example, ref. [29] constructed a multi-task fusion system by combining the spatial information of facial features with the temporal sequence of yawning behaviors, achieving recall rates of 84–85% on the YawDD, MiraclHB, and DEAP datasets. Similarly, ref. [30] focused exclusively on temporal characteristics, tracking the time-series variations in the pupil center and the horizontal distance between the eyes using occlusion-based standards, and achieved 89% accuracy with an SVM classifier on public benchmark datasets. Building upon these efforts, ref. [31] proposed a spatio-temporal graph convolutional network (ST-GCN) that employs a dual-stream architecture to capture both the spatial structural features of facial landmarks and the temporal dynamics of fatigue behaviors, fusing first- and second-order spatio-temporal information. This approach achieved average accuracies of 93.4% and 92.7% on the YawDD and NTHU-DDD datasets, respectively, demonstrating a deep synergy between spatial and temporal feature learning. In addition, ref. [32] centered on temporal features supported by spatial information extracted via Dlib, analyzing the time-series pattern of blink duration, and obtained 94.2% accuracy in open-eye state detection on the IMM face dataset after occlusion preprocessing. Ref. [33] explicitly adopted a spatial–temporal dual-feature framework, designing two model variants: one used LSTM to extract temporal features of fatigue behavior combined with Dlib and a linear SVM to achieve 79.9% accuracy, while the other combined CNN-based spatial extraction with LSTM temporal modeling and a Softmax classifier, achieving 97.5% accuracy, though with higher computational cost due to the large multi-feature matrices. Likewise, ref. [34] employed MTCNN and Dlib to extract spatial features of facial, mouth, and eye regions, combining them with temporal sequences at constant frame rates to compute fatigue states, yielding 98.81% accuracy on the YawDD and NTHU-DDD datasets and achieving fundamental spatio-temporal fusion. Ref. [35] further extracted facial keypoints using MTCNN and refined localization accuracy via Dlib, transforming each frame’s spatial feature vector into temporal sequences input to an LSTM to model fatigue evolution, reaching 88% and 90% accuracy on the YawDD and self-constructed datasets, respectively.

Real-time driver drowsiness detection remains a challenging research problem, and several studies have made progress by optimizing detection speed and adapting models to real-time data streams. Ref. [36] proposed a hybrid architecture combining Haar cascades with a lightweight CNN, validated through fivefold cross-validation on the UTA-RLDD dataset, achieving a detection speed of 8.4 frames per second. When tested on a custom real-time video dataset collected from 122 participants, the model achieved 96.8% accuracy after hyperparameter tuning with batch sizes of 200 and 500 epochs. Similarly, ref. [28] developed an entropy-based real-time analysis system using an improved YOLOv3-tiny CNN for rapid face-region detection, coupled with Dlib-based extraction of the spatial coordinates of facial triangles and temporal sequences of facial landmarks to construct fatigue-related feature vectors. The proposed system achieved speeds exceeding 20 frames per second and an accuracy of 94.32% on the YawDD dataset. In another effort, ref. [37] established a four-class real-time detection framework using real-world data from 223 participants, selecting 245 five-minute videos from 10 subjects with a 3:1 training-validation split. Trained on real-time data, this framework achieved a maximum accuracy of 64%, highlighting the difficulties in maintaining robustness and accuracy under natural driving conditions.

To enhance robustness and balance model performance under complex driving environments, researchers have explored architectural optimization and multi-feature fusion strategies. Ref. [38] addressed challenges such as eyeglass reflections and variable illumination by proposing a 3DcGAN + TLABiLSTM spatio–temporal fusion architecture, which achieved 91.2% accuracy on the NTHU-DDD dataset and improved adaptability to environmental variations. Meanwhile, ref. [39] introduced a multi-feature fusion system that integrates Haar-like features, 68 facial landmarks, PERCLOS, and MAR metrics, combined with VGG16 and SSD algorithms, achieving 90% accuracy on the NTHU-DDD dataset while effectively mitigating the effects of lighting variation and eyeglass interference. Regarding model lightweighting and generalization, ref. [40] compared multiple deep networks including Xception, ResNet101, InceptionV4, ResNeXt101, and MobileNetV2, revealing that MobileNetV2 significantly reduces computational demand while maintaining 90.5% accuracy, whereas a Weibull-optimized ResNeXt101 achieved 93.80% accuracy and 84.21% generalization accuracy on the NTHU-DDD dataset, offering a practical balance between model efficiency and generalization. Finally, ref. [41] optimized temporal fatigue-state modeling on the RLDD dataset using an HM-LSTM network, improving accuracy from 61.4% (baseline LSTM) to 65.2%, thus enhancing model generalization capacity for real-world driving fatigue recognition.

Overall, driver fatigue detection has transitioned from single facial spatial cues to a framework that deeply fuses spatial and temporal features, jointly optimizing real-time performance and robustness. Coordinated spatiotemporal modeling has become the core of behavior-driven analysis, while advances in the YOLO family and lightweight backbones provide the critical support required for real-time deployment in driving scenarios. At the same time, multi-feature fusion and adaptation to complex environments remain key challenges for future work—and they directly inform the model design and performance optimizations pursued in this study.

## 3. Methodology

### 3.1. Face Detection Module Based on MG-YOLOv8

This paper proposes a face detection framework based on the improved MG-YOLOv8. Based on the YOLOv8 model, it optimizes gradient propagation paths using the C2f module and incorporates the SPPF (Spatial Pyramid Fast Pooling) module for multiscale feature fusion, significantly enhancing detection accuracy and real-time performance in complex scenarios. (1) Integration of MLCA (Mixed Local Channel Attention), which strengthens feature representation of key facial regions through dynamic weight allocation and suppresses complex background interference; (2) Introduction of the GELAN design concept, employing its core unit RepNCSPELAN4 to enhance multi-branch feature aggregation and re-parameterized representation, thus improving robustness under occlusion and scale variation; (3) Use of the EIoU loss function instead of the traditional CIoU, optimizing small object bounding box regression accuracy through the introduction of aspect ratio decoupling. The model is trained end-to-end in a large-scale multiscene face dataset, achieving a 2% improvement in the accuracy of the location of the face compared to the baseline model.

**Figure 1:**
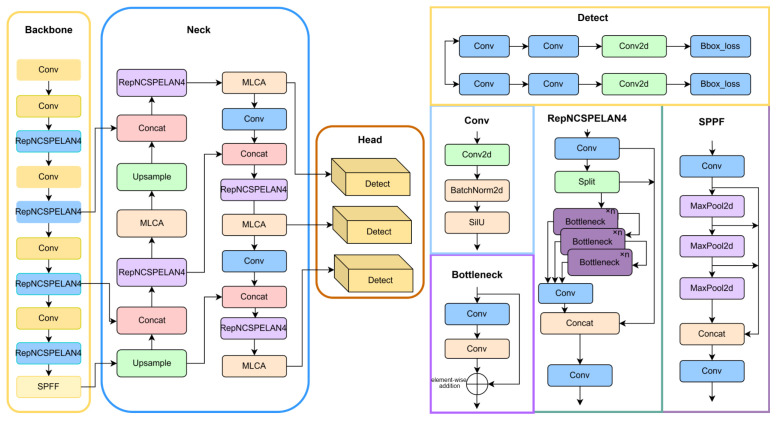
MG-YOLOv8 Network Architecture Diagram.

As shown in Figure 1, the network architecture of MG-YOLOv8 replaces each stage of the original C2f with a Conv–RepNCSPELAN4–Conv stack in the backbone while retaining the SPPF module, forming a GELAN-style ELAN/Rep multi-branch aggregation structure. In the neck (PAN-FPN), MLCA modules are inserted after each upsampling-Concat operation in the P5 → P4 and P4 → P3 paths, as well as after two bottom-up Concat operations, to enhance cross-scale information selection. The head maintains the three-scale detection branches of YOLOv8 unchanged, with the bounding-box regression loss replaced by EIoU for improved precision.

#### 3.1.1. Mixed Local Channel Attention (MLCA)

The existing channel attention mechanisms (e.g., SE, ECA) primarily focus on global relationships between channels while neglecting spatial information, resulting in the loss of local key features during the compression process. However, pixel-level spatial attention mechanisms such as CA and CBAM suffer from heatmap redundancy issues. To effectively incorporate spatial information, the local SE (LSE) divides the feature map into blocks, resembling multiple stacked local SEs. However, too many blocks increase the number of parameters. Reducing channel dimensions reduces LSE parameters and computational complexity but can lead to loss in accuracy. Therefore, this paper proposes a Hybrid Local Channel Attention (MLCA) mechanism to enhance local feature representation with low computational cost.

**Figure 2:**
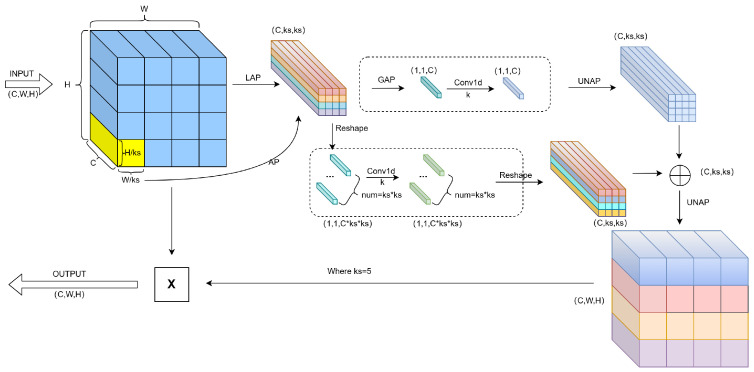
Schematic Diagram of MLCA Algorithm. Different colors indicate various feature types and operations for visual distinction.

The main process of MLCA is shown in Figure 2. MLCA employs a dual-branch structure to integrate global and local information. First, the input feature map undergoes local average pooling (LAP) to produce a 1*C*ks*ks vector, extracting local features. Then, Global Average Pooling (GAP) captures global information, which is fused with the features through a Conv1D operation. The kernel size *k* is logarithmically related to the channel number *C*, enhancing cross-channel interactions locally. Finally, an Unpooling (UNAP) operation restores the feature map size, achieving fusion of global and local information. The selection of k [42] is determined by the following formula:

(1) $$k=\varphi \left(C\right)=|\frac{\log2(C)}{\gamma }+\frac{b}{\gamma }{|}_{odd}$$

where ${\left|t\right|}_{odd}$ denotes the nearest odd integer to *t*; *C* represents the number of channels in the input feature map, as illustrated in Figure 2; k is the convolution kernel size, and γ and b are hyperparameters with a default value of 2.

#### 3.1.2. Generalized Efficient Layer Aggregation Network (GELAN)

The Generalized Efficient Layer Aggregation Network (GELAN) is a task-oriented advanced network architecture designed for lightweight and efficient object detection by effectively planning gradient paths. The input tensor typically consists of a batch of images with predefined channels, height, and width [43]. GELAN captures high-level semantic information and fundamental details required for object detection. In the backbone network, the input tensor undergoes multiple convolutions, potentially increasing the channel number while reducing the spatial dimensions (height and width) of the feature map. This hierarchical feature extraction enables the model to process multi-scale features.

**Figure 3:**
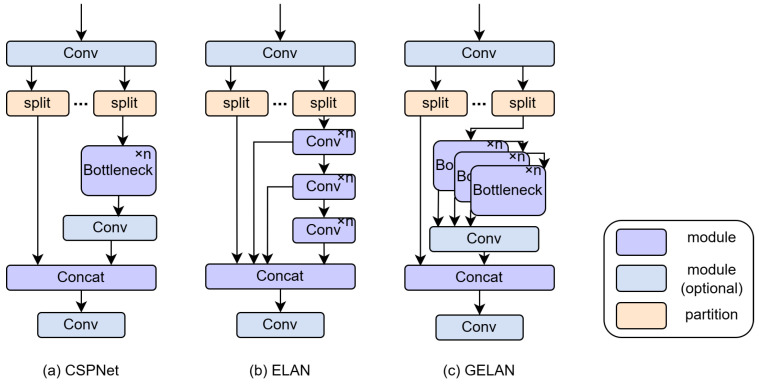
GELAN Architecture Diagram [47].

The GELAN structure, shown in Figure 3, integrates the design principles of CSPNet [44] and ELAN [45]. CSPNet splits input through transition layers, computes features separately, and then merges them, while ELAN employs stacked convolutions to gradually integrate features. GELAN inherits CSPNet’s split-recombine property and combines ELAN’s layered convolutions to improve network adaptability, allowing selection of computational blocks as needed.

Additionally, key optimizations in GELAN include upsampling to improve feature map resolution and fusion with early-layer features to enhance fine-grained information and spatial connectivity, thereby improving object localization accuracy. Spatial Pyramid Pooling (SPP) and RepNCSPELAN4 further optimize feature representation, enhancing the network’s ability to handle multiscale features.

The detection module in GELAN receives feature maps from different detection layers and generates class confidence and bounding-box predictions. During inference, the anchor boxes and the strides are dynamically adjusted to ensure precise object detection. Its efficient inference capabilities and scalability make it suitable for applications in autonomous driving, medical imaging, and surveillance systems [46].

#### 3.1.3. Loss Function (EIoU)

In the conventional YOLOv8 architecture, the loss function is primarily composed of three components: the object confidence loss, the classification loss, and the regression loss. Among these, both the object confidence loss and the classification loss are typically computed using Binary Cross-Entropy (BCE), while the regression loss initially adopts the CIoU Loss [48]. The design of CIoU is fundamentally based on the Intersection over Union (IoU) metric, a classical indicator in object detection tasks used to evaluate the degree of overlap between the predicted bounding box and the ground-truth box. Specifically, IoU is defined as the ratio between the intersection area and the union area of the predicted and ground-truth boxes. The IoU can be mathematically expressed as follows:

(2) $$IoU\left(A,B\right)=\frac{\left|A\cap B\right|}{\left|A\cup B\right|}$$

The CIoU loss considers three critical geometric factors: the overlap area, the distance between the centers, and the aspect ratio. Given a predicted bounding box b a ground truth box b_gt_, the CIoU loss is defined as follows:

$$L_{\mathrm{CIoU}\;}=1-IoU+\frac{\rho^{2}\left(b,b_{gt}\right)}{C^{2}}+\alpha v$$

where *b* and $b_{gt}$ represent the centers of the predicted and ground truth bounding boxes, respectively, $\rho^{2}\left(b,b_{gt}\right)$ denotes the Euclidean distance between these centers, and is the diagonal length of the smallest enclosing box that contains both the predicted and ground truth boxes and where $v=\frac{4}{\pi^{2}}{\left(\arctan\frac{w^{gt}}{h^{gt}}-\arctan\frac{w}{h}\right)}^{2},\alpha =\frac{v}{(1-IoU)+v}$ is used to measure the difference in aspect ratio.

The gradient of width *w* and height *h* with respect to *v* is given by the following formula:

$$\frac{\partial v}{\partial w}=\frac{8}{\pi^{2}}\left(\arctan\frac{w^{gt}}{h^{gt}}-\arctan\frac{w}{h}\right)\cdot \frac{h}{w^{2}+h^{2}}$$

$$\frac{\partial v}{\partial h}=-\frac{8}{\pi^{2}}\left(\arctan\frac{w^{gt}}{h^{gt}}-\arctan\frac{w}{h}\right)\cdot \frac{w}{w^{2}+h^{2}}$$

The CIoU loss function enhances localization accuracy in object detection by comprehensively considering geometric factors, particularly optimizing the aspect ratio. Although previous studies [48] have demonstrated that CIoU loss achieves faster convergence and higher detection accuracy compared with earlier loss functions, the last term in the CIoU formulation remains ambiguously defined, which may slow down convergence. Therefore, in the proposed MG-YOLOv8 framework, the EIoU loss function is adopted instead. Unlike CIoU, EIoU directly regresses the actual width and height of the predicted bounding box rather than relying solely on aspect ratio optimization. This approach effectively reduces the negative impact of aspect ratio uncertainty on loss computation, leading to more stable and consistent regression performance. The definition of EIoU [49] is given as follows:

(3) $$L_{EIoU}=L_{IoU}+L_{dis}+L_{asp}=1-IoU+\frac{\rho^{2}\left(b,b^{gt}\right)}{{\left(h^{c}\right)}^{2}+{\left(w^{c}\right)}^{2}}+\frac{\rho^{2}\left(w,w^{gt}\right)}{{\left(w^{c}\right)}^{2}}+\frac{\rho^{2}\left(h,h^{gt}\right)}{{\left(h^{c}\right)}^{2}}$$

where $w^{c}$ and $h^{c}$ represent the height and width of the minimum enclosing rectangle covering both the predicted and ground truth boxes; *w* and *h* are the width and height of the predicted box, while $w^{gt}$ and $h^{gt}$ represent the width and height of the ground truth box.

### 3.2. Fatigue Detection Module

**Figure 4:**
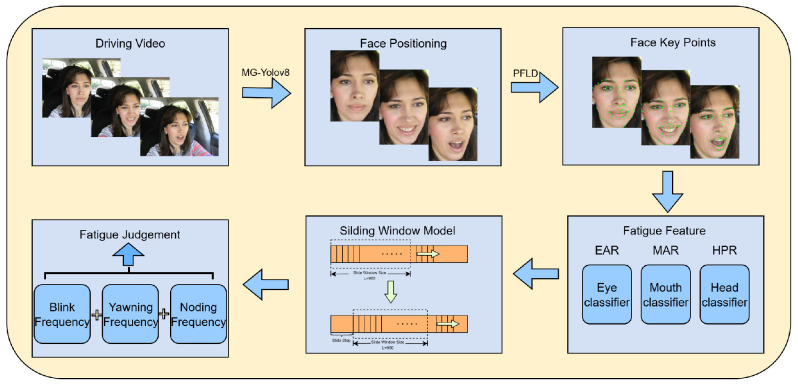
Overall System Framework Diagram.

Using the face detection results from MG YOLOv8, the system employs the lightweight PFLD (Practical Facial Landmark Detector) model to extract 68 key points on the face. It then calculates parameters such as EAR, MAR, and HPR in real time. To improve environmental adaptability, this paper presents a novel sliding window model that uses a dynamic threshold adjustment strategy and an Exponential Weighted Moving Average (EWMA) algorithm. This model performs EWMA smoothing and dynamic threshold adjustment within each sliding window to reduce short-term noise and individual differences. The model then calculates temporal features, such as BF, PERCLOS, YF, and NF; performs normalization and weighted fusion; and generates a comprehensive fatigue index to trigger graded warnings. Through real-time signal processing and feature fusion within the sliding window, this algorithm achieves highly robust detection of fatigue behavior in complex scenarios. Figure 4 shows the complete system framework, and subsequent sections will introduce the detailed implementation.

#### 3.2.1. Facial Landmark Detection and Feature Extraction

**Figure 5:**
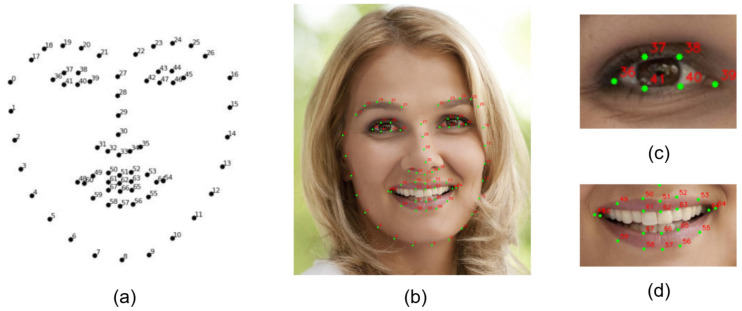
Facial Key Points Schematic Diagram. (**a**) Shows the index layout of the 68 facial landmarks; (**b**) displays the detected landmark points on the full face; (**c**) presents a close-up of the eye region with labeled key points; and (**d**) shows the key points located in the mouth region.

This paper uses a lightweight PFLD model to precisely localize facial features. The 68-keypoint coordinate system output (Figure 5) accurately maps the eye, mouth, and facial contour regions. The system first processes each frame of the video sequence and uses the distribution of key points to perform clustering analysis and region segmentation on the face. This process achieves precise localization of the eyelid and mouth contours. The eye aspect ratio (EAR) is defined based on the spatial layout of the eye key points as the ratio of the Euclidean distances between the vertical and horizontal feature points of the eye. The specific calculation formula is as follows:

(4) $$\mathrm{EAR}=\frac{∥P_{37}-P_{41}∥+∥P_{38}-P_{40}∥}{2∥P_{36}-P_{39}∥}$$

Similarly, MAR is another metric that reflects the yawning behavior based on the degree of mouth opening, and it is calculated as follows:

(5) $$\mathrm{MAR}=\frac{∥P_{50}-P_{58}∥+∥P_{52}-P_{56}∥}{2∥P_{54}-P_{48}∥}$$

For both EAR and MAR, $P_{i}=\left(x_{i},y_{i}\right)$ denotes the coordinate of the *i*-th landmark, and $∥P_{a}-P_{b}∥$ denotes the Euclidean distance between two landmarks.

PERCLOS is a metric used to quantify the proportion of time during which a driver’s eyes remain closed within a given observation period. According to the P80 standard, an eye is considered closed when the eyelid covers more than 80% of the eyeball area; otherwise, it is classified as open. However, due to individual biophysical variations, the P80 threshold is not universally applicable. To improve the accuracy of PERCLOS estimation, the eye-closure process is refined across multiple image frames, and the ratio of closed-eye frames to the total number of frames is computed. This approach provides a more precise reflection of the driver’s fatigue state. The calculation formula is expressed as follows:

(6) $$PERCLOS_{new}=\frac{\sum_{n=1}^{T}f_{n}}{T}\times 100\%$$

where *T* represents the total number of frames within a sliding window, and $f_{n}$ denotes the eye state at frame n. When the eyes are determined to be open, $f_{n}$ is assigned a value of 0; conversely, when the eyes are determined to be closed, $f_{n}$ is assigned a value of 1. In addition to blinking and yawning, forward and backward head movements also serve as important indicators of fatigue. Therefore, head postures in the pitch, yaw, and roll directions are commonly employed as fatigue evaluation metrics. In this study, the concept of Head Pose Ratio (HPR) is introduced, which maps 2D facial images onto a 3D model to assess head posture. The corresponding Euler angle transformation is expressed as follows:

(7) $$HPR=\left(\begin{array}{c} \alpha_{x}\\ \alpha_{y}\\ \alpha_{z} \end{array}\right)=\left(\begin{array}{c} a\tan2(\lambda_{21},\lambda_{22})\\ a\tan2(-\lambda_{20},\sqrt{\lambda_{21}^{2}+\lambda_{22}^{2}})\\ a\tan2(\lambda_{10},\lambda_{00}) \end{array}\right)$$

The corresponding rotation matrix is expressed as shown in Equation (8), where θ, φ and ϕ represent the pitch, yaw, and roll angles, respectively. The HPR can be derived from the 3D model of α using the parameter λ defined in Equation (7). According to anatomical and medical knowledge, the normal ranges of human head pitch, roll, and yaw angles are (−60.4°, 69.6°), (−41°, 36.3°), and (−75°, 75°), respectively. If the amplitude of head movement exceeds 70% of these ranges, it is considered an abnormal postural variation [50]. Within a given time interval, when the actual change in Euler angles exceeds the HPR threshold, the corresponding head posture can be regarded as a nodding motion, and its frequency can be used as an indicator for fatigue detection.

(8) $$\begin{array}{c} R=\left(\begin{array}{ccc} \lambda_{00} & \lambda_{01} & \lambda_{02}\\ \lambda_{10} & \lambda_{11} & \lambda_{12}\\ \lambda_{20} & \lambda_{21} & \lambda_{22} \end{array}\right)=\\ \left(\begin{array}{ccc} \cos\varphi \cos\phi +\sin\varphi \sin\theta \sin\phi & \cos\theta \sin\varphi & -\cos\varphi \cos\phi +\sin\varphi \sin\theta \cos\phi \\ -\sin\varphi \cos\phi +\cos\varphi \sin\theta \sin\phi & \cos\theta \cos\varphi & \sin\varphi \cos\phi +\cos\varphi \sin\theta \cos\phi \\ \cos\theta \sin\phi & -\sin\theta & \cos\theta \cos\phi \end{array}\right) \end{array}$$

#### 3.2.2. Innovative Sliding Window Model

To enable the model to effectively adapt to individual physiological differences and the temporal evolution patterns of driver fatigue behaviors, this study proposes an innovative sliding window model that integrates a dynamic threshold adjustment mechanism with an exponential weighted moving average (EWMA) strategy. The proposed model enables stable and accurate assessment of fatigue states across different drivers. For clarity and reproducibility, the key steps of the proposed sliding window approach are summarized in Algorithm 1.
**Algorithm 1** Innovative Sliding Window Algorithm for Fatigue Feature Extraction.1:**Input:** Time sequences of EAR, MAR, and HPR over *T* frames; EWMA coefficient α; window size *S*; stride *s*; minimum segment durations $L_{y},L_{n}$; thresholds $thr_{yawn},thr_{nod},thr_{ear}$; hysteresis *h*.2:**Output:**BF, YF, NF, and PERCLOS.3:**Step 1:** Apply EWMA smoothing to EAR, MAR, and HPR sequences.4:**Step 2:** Initialize the adaptive EAR threshold and determine eye states (*open*/*closed*) with hysteresis control.5:**Step 3:** Divide the signal into overlapping sliding windows of length *S* and stride *s*.6:**Step 4:** Within each window, compute:       BF: number of eye-closing transitions;    YF: number of continuous segments where $MAR>thr_{yawn}$ for at least $L_{y}$ frames;       NF: number of continuous segments where $HPR>thr_{nod}$ for at least $L_{n}$ frames;       PERCLOS: proportion of frames with closed eyes.7:**Step 5:** Output temporal fatigue features {BF,YF,NF,PERCLOS}.

The model uses a sliding window as the temporal processing unit and incorporates individualized dynamic threshold settings and EWMA smoothing strategies within the window. This forms a unified detection framework that combines “trend modeling + state determination.” First, during the initial detection phase, the system calculates the average EAR value of the previous two frames and compares it with a predefined baseline threshold (typically set to 0.25). It then takes the smaller value as the personalized initial EAR threshold to accommodate individual physiological differences. Then, during processing of each frame, EWMA smoothing calculations are performed on features such as EAR, MAR, and HPR to suppress interference from short-term fluctuations in state determination. The basic formula for EWMA is as follows:

(9) $$EWMA_{t}=\alpha \cdot Value_{t}+\left(1-\alpha \right)\cdot EWMA_{t-1}$$

where $Value_{t}$ represents the raw data value at the current time step and α is the smoothing coefficient set to 0.2.

Within the scope of the sliding window, the model compares the smoothed results of each frame against a threshold to identify behavioral events (e.g., blinking, yawning, nodding) that significantly exceed or fall below the threshold for a certain period of time. Specifically, the model compares the exponential weighted moving average (EWMA) values of eye aspect ratio (EAR), mouth aspect ratio (MAR), and head posture ratio (HPR) with the thresholds shown in Table 1 on a frame-by-frame basis. When EAR remains below the threshold for a certain number of frames, it is identified as a blink. When MAR remains above the threshold, it is identified as a yawn. When HPR exceeds the threshold and persists for a certain period of time, it is identified as a nodding event.

Based on the comparison results, calculate the blink frequency (BF), yawn frequency (YF), and nod frequency (NF) in a video clip using the following formulas:

(10) $$\begin{array}{c} BF_{i}=\sum_{t=1}^{T}Blink_{(i-1)S+t},\;YF_{i}=\sum_{t=1}^{T}Yawn_{(i-1)S+t},\;NF_{i}=\sum_{t=1}^{T}Nod_{(i-1)S+t},\\ \mathrm{where}\;i=1,2,3,\ldots ,n \end{array}$$

where $BF_{i}$, $YF_{i}$ and $NF_{i}$ represent the frequency of fatigue behavior in the i-th sliding window, i denotes the index of the current sliding window, T is the total number of frames within a sliding window, S is the sliding step size, t indicates the frame position within the current window, and n represents the total number of sliding windows.

**Figure 6:**
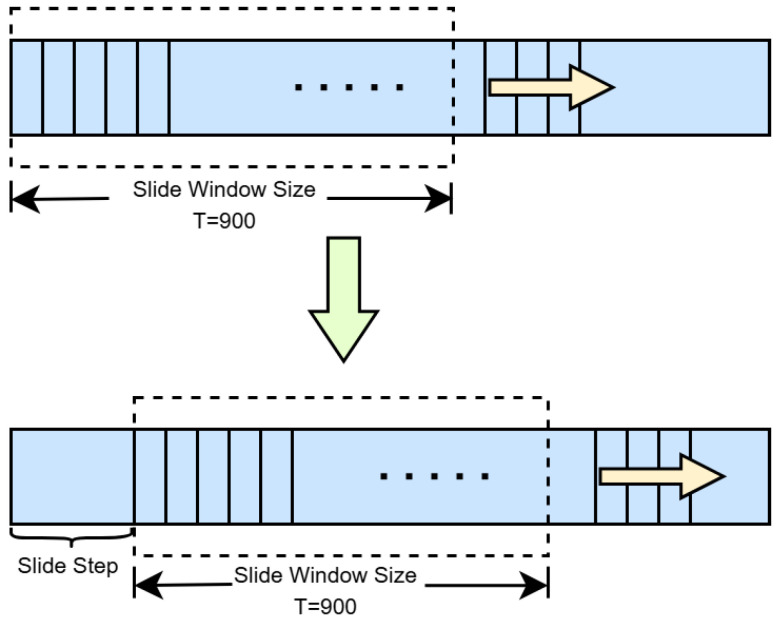
Schematic Diagram of the Sliding Window Model.

In this study, the sliding window mechanism was optimized experimentally to balance real-time performance and detection sensitivity. For long-term fatigue assessment, a large window size (T = 900) was employed to capture stable behavioral trends, while for real-time fatigue warning, a shorter step size (S = 150) was adopted to enhance response speed. In cases where the total number of video frames was not an integer multiple of T, the remaining frames were normalized by the ratio of remaining frames to T for fatigue parameter calculation. This strategy ensures detection accuracy while reducing computational latency and improving the system’s adaptability to dynamic driving environments. The schematic diagram of the sliding window model is shown in Figure 6.

In summary, the temporal modeling components play complementary roles in fatigue detection. The sliding window mechanism balances timeliness and stability, enabling the sensitive capture of transient behaviors such as brief eye closures or subtle head nods. The EWMA smoothing mechanism effectively suppresses high-frequency fluctuations caused by lighting variations, facial micro-expressions, or sensor noise, making the fatigue-related feature curves smoother and more distinguishable. Meanwhile, the dynamic threshold and hysteresis control ensure adaptive and consistent decision-making across different individuals and varying environmental conditions. Combined, these components form a temporal analysis framework that integrates robustness, sensitivity, and generalization, allowing for precise tracking of fatigue feature evolution while avoiding overreaction to short-term noise. This integration ultimately enables high-accuracy, low-latency, and robust fatigue detection under realistic driving conditions.

#### 3.2.3. Comprehensive Fatigue State Decision-Making Algorithm

**Figure 7:**
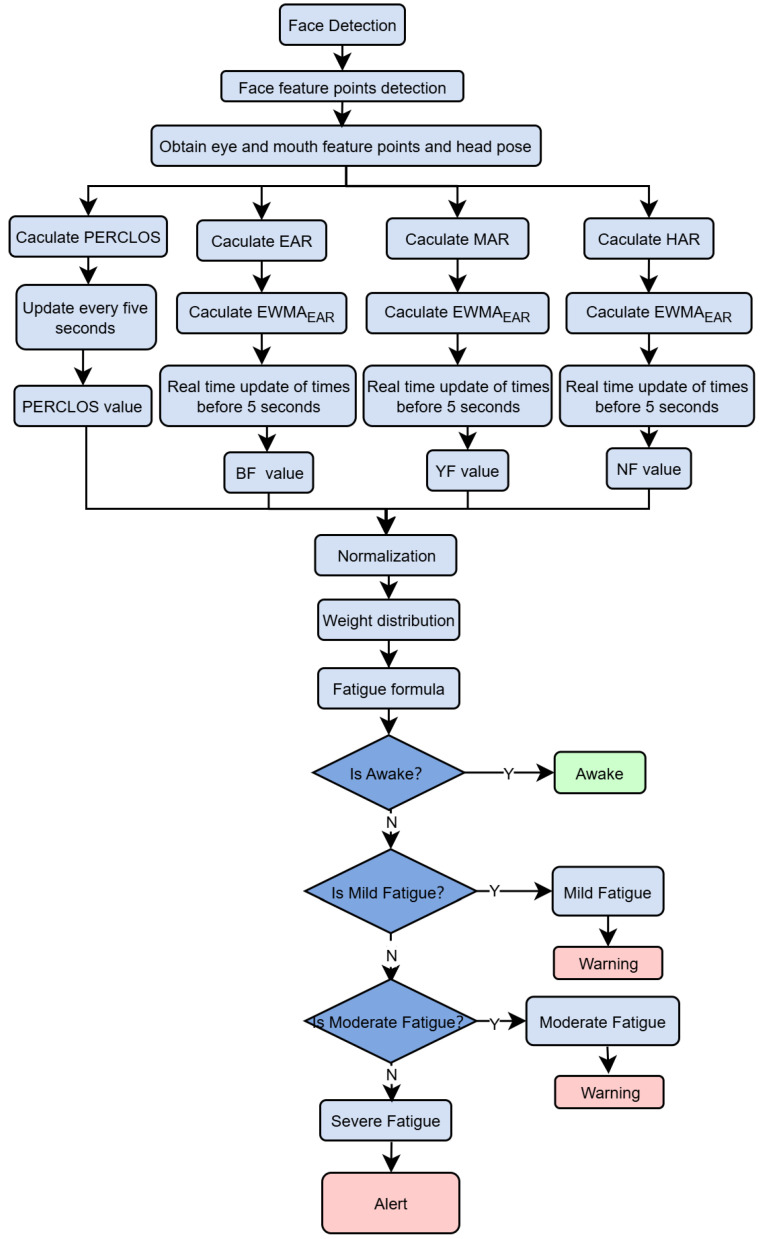
Fatigue Detection Algorithm Framework.

To achieve a comprehensive evaluation of the driver’s fatigue state, the system first identifies and records fatigue-related behavioral events—such as blinking, yawning, and nodding—within each sliding window. Subsequently, the proposed integrated fatigue assessment algorithm is applied for further analysis. Specifically, the algorithm extracts four key fatigue-related features from each window: blink frequency (BF), PERCLOS, yawn frequency (YF), and nod frequency (NF). Figure 7 below illustrates the overall process of the proposed fatigue detection algorithm.

Due to differences in numerical scales and distribution ranges among various indicators, the system uses an adaptive normalization formula to compress the indicators into the (0, 1) interval, unifying the measurement standards and facilitating subsequent weighted fusion and fatigue level classification. The normalization formula is shown below:

(11) $$F_{i}=\frac{X_{i}-X_{\left(i,0\right)}}{X_{\left(i,m\right)}-X_{\left(i,0\right)}},i=1,2,3,4;$$

For the currently monitored values (such as BF, PERCLOS, YF, and NF), $X_{\left(i,0\right)}$ represents the baseline value and $X_{\left(i,m\right)}$ represents the maximum reference value of each parameter (see Table 2). If the monitored value $X_{i}$ exceeds $X_{\left(i,m\right)}$ or is lower than $X_{\left(i,0\right)}$, it can be classified as 1 or 0, respectively, to ensure that the result is within the (0, 1) interval.

After normalization, these four parameters are combined into a fatigue evaluation index F, which is calculated as follows:

(12) $$F=\sum_{i=1}^{4}W_{i}F_{i},\sum_{i=1}^{4}W_{i}=1$$

where $W_{i}$ denotes the weight associated with the corresponding fatigue parameter $F_{i}$. To verify the rationality of the proposed weight configuration, an ablation experiment on feature fusion weights was conducted using the public dataset described in Section 4.4. The detection and temporal modules were kept unchanged, while only the weighting vector $W_{i}$ for multi-feature fusion was adjusted. Twelve candidate weighting schemes were designed, and all were evaluated on the same dataset with average accuracy as the primary performance metric. The experimental results are presented in Table 3.

As shown in Table 3, Group G4 (($W_{1}$ = 0.10, $W_{2}$ = 0.40, $W_{3}$ = 0.30, $W_{4}$ = 0.20)) achieved the highest accuracy of 94.50%, outperforming both the uniformly weighted configuration G1 (91.13%) and other comparable combinations such as G3, G5, and G8. This indicates that a weighting strategy dominated by PERCLOS, complemented by YF and NF, and denoised by BF, yields more robust performance under challenging conditions such as illumination variations and mild occlusions. In contrast, when only a single feature channel was retained (G9–G12), the performance dropped significantly, and even dual-feature or triple-feature configurations (e.g., G6 and G7) failed to match the effectiveness of the four-feature fusion scheme. These results further validate the superiority of the proposed multi-feature fusion approach over single-feature methods. The adopted weight configuration also demonstrated consistent performance in subsequent validation experiments, suggesting good generalizability. Nevertheless, future studies are encouraged to fine-tune the weight distribution according to specific application scenarios, such as variations in camera placement across vehicle models or physiological differences among driver groups with different age characteristics.

After determining the optimal feature weight configuration, a comprehensive fatigue evaluation metric F was computed based on the selected weights. The value of F ranges from 0 to 1, where a higher value indicates a greater degree of driver fatigue. According to different ranges of F, the driver’s fatigue state can be classified into four levels. The detailed logic for fatigue state determination and the corresponding alert output rules are presented in Algorithm 2.
**Algorithm 2** Comprehensive Fatigue Assessment Algorithm.  1:**Input:** fatigue index parameter PERCLOS value and F value  2:**Output:** fatigue level  3:**if** PERCLOS >0.4 **then**  4:      **Warning:** The driver is sleeping  5:**else**  6:      **if** F<0.25 **then**  7:           Awake state  8:      **else if** F≥0.25 and F<0.45 **then**  9:           Mild fatigue state10:      **else if** F≥0.45 and F<0.65 **then**11:           Moderate fatigue state12:      **else if** F≥0.65 and F<1.0 **then**13:           Severe fatigue state14:      **end if**15:**end if**

## 4. Experiment Analysis

### 4.1. Experiment Environment

To validate the effectiveness of the proposed method, an experimental platform was established using Windows 11 as the operating system and PyTorch 1.13.1 as the deep learning framework. YOLOv8s was employed as the baseline network model. The specific configuration of the experimental environment is detailed in Table 4.

Consistent hyperparameters were applied throughout the training process for all experiments. Table 5 shows the exact hyperparameters used during training.

### 4.2. Dataset Selection and Preprocessing

1. WIDER FACE Dataset [51]: This benchmark dataset for face detection consists of 32,203 images with 393,703 labeled faces, exhibiting significant variations in scale, pose, and occlusion. The dataset is categorized into 61 scene types and is split into 40% for training, 10% for testing, and 50% for validation, enabling a comprehensive evaluation of model performance across diverse scenarios.
2. YAWDD Dataset [52]: This dataset is specifically designed for fatigue driving detection, capturing facial features of drivers in real-world driving environments across different genders, ethnicities, and eyewear conditions during activities such as talking, singing, remaining silent, and yawning. The YAWDD dataset consists of two video subsets, both recorded under realistic and varying lighting conditions. The first subset (322 videos) was captured with a camera mounted below the rearview mirror, covering three driving states: normal driving (silent), talking or singing while driving, and yawning while driving, with each participant contributing 3–4 videos. The second subset (29 videos) was recorded with a dashboard-mounted camera, where each participant provided one video encompassing all mouth movement states.

In this study, the WIDER FACE dataset was employed as both the training and validation set to train and evaluate the performance of the improved YOLOv8-based face detection model. This dataset encompasses a wide range of facial samples under varying lighting conditions, poses, occlusions, and resolutions, thereby enhancing the model’s feature learning capability and comprehensively verifying its robustness and accuracy in complex scenarios. Meanwhile, the YAWDD dataset was selected as the primary benchmark for evaluating the proposed fatigue detection method, aiming to assess the effectiveness of the multi-feature fusion and temporal modeling strategies in real driving environments. The YAWDD dataset includes diverse driver facial behaviors and expressions—such as blinking, yawning, and talking—which enable a thorough evaluation of the model’s performance in fatigue behavior recognition, dynamic feature extraction, and robustness under complex environmental conditions.

### 4.3. Performance of MG-YOLOv8 in Face Detection

#### 4.3.1. Evaluation Metrics

To evaluate the performance of the trained MG-YOLOv8 model, the accuracy of the detection model is assessed using precision (P) and mean average precision (mAP). Additionally, the model speed is evaluated on the basis of the number of parameters (Params) and floating-point operations (GFLOPS).

Precision (P) measures the accuracy with which the model identifies true positives among all positive predictions, reflecting its capability for precise localization of target instances. As a core metric in object detection, high precision is particularly critical for driver monitoring systems, as it ensures accurate identification of fatigue states, reduces false alarms, and enhances the overall reliability and practical applicability of the system.

(13) $$Precision=\frac{TP}{TP+FP}$$

Here, the variable TP denotes the number of true positive samples correctly predicted as positive, while FP represents the number of false positive samples that were incorrectly predicted as positive.

Mean Average Precision (mAP) is a commonly used metric for evaluating the performance of object detection algorithms. It represents the mean value of the Average Precision (AP) across all object categories. For a single category, AP is defined as the area under the Precision–Recall (PR) curve within the recall range of 0 to 1, which comprehensively reflects the model’s precision performance under different recall requirements. In calculating mAP, the predicted bounding boxes are first sorted according to their confidence scores. Then, the AP value for each category is computed under different Intersection over Union (IoU) thresholds. Finally, the mAP is obtained by averaging the AP values across all categories. The corresponding calculation formula is expressed as follows:

(14) $$\mathrm{AP}=\int_{0}^{1}P\left(R\right)dr$$

(15) $$mAP=\frac{\sum_{0}^{N}AP_{n}}{N}$$

Here, N denotes the total number of object categories, and represents the Average Precision (AP) corresponding to the N-th category.

mAP@0.5 refers to the mean Average Precision computed for all object categories at an Intersection over Union (IoU) threshold of 0.5.

Params refers to the total number of trainable parameters in the model, including the weights and biases of convolutional layers and other learnable components. The number of parameters directly affects the model’s size and structural complexity.

GFLOPs denotes the number of floating-point operations, which serves as a measure of the model’s computational complexity and efficiency. A higher FLOP value indicates greater model complexity and computational demand.

#### 4.3.2. Training and Performance Analysis of the Object Detection Model

To evaluate the performance improvements achieved through backbone optimization, the integration of the MLCA module, and the adoption of the EIoU loss function, a series of comparative experiments were conducted. Key performance metrics—including Precision (P), mean Average Precision (mAP), the number of model parameters (Params), and computational complexity (GFLOPs)—were used to comprehensively assess the original YOLOv8s model and the improved version. The detailed ablation results on the WIDER FACE dataset are presented in Table 6.

As shown in Table 6, incorporating the RepNCSEPLAN4 module optimized the network structure, resulting in a reduction of approximately 29.1% in the number of parameters and about 29.7% in computational complexity. However, a slight decrease in mAP@0.5 was observed. The introduction of the MLCA module had minimal impact on model parameters and computational cost, yet it significantly improved the precision (P) from 0.889 to 0.906. Employing the EIoU loss function led to a moderate increase in precision from 0.889 to 0.893 without adding extra computational burden. Combining the RepNCSEPLAN4 and MLCA modules further enhanced the precision to 0.908 while maintaining lightweight characteristics, with only 20.2 GFLOPs and 7.90 M parameters. Similarly, integrating RepNCSEPLAN4 with EIoU achieved a balance between accuracy improvement and model compactness. The combination of MLCA and EIoU raised the precision to 0.909, leveraging the complementary strengths of the two modules for enhanced performance. Overall, the improved model that integrates RepNCSEPLAN4, MLCA, and EIoU outperformed the baseline YOLOv8s in terms of detection accuracy, computational efficiency, and parameter compactness. On the same dataset, the fully integrated model achieved a mAP@0.5 of 0.818, with approximately 29.2% fewer parameters, 29.7% lower computational complexity, and the highest precision (P) of 0.910.

This study compares the performance of the proposed method with other face detection approaches on the WIDER FACE dataset. The comparative results are presented in Table 7.

As shown in Table 7, the proposed MG-YOLOv8 model achieves competitive overall performance compared with several mainstream face detection algorithms. Specifically, relative to the baseline YOLOv8s, MG-YOLOv8 attains a higher precision (*p* = 0.910) while maintaining a lower computational complexity (20.1 GFLOPs) and fewer parameters (7.88 M), with only a slight decrease in mAP@0.5. When compared with traditional detectors such as SSD and Faster R-CNN, MG-YOLOv8 demonstrates superior accuracy and inference efficiency, exhibiting notable advantages in model lightweighting and real-time performance. These results indicate that the optimization strategies—including backbone modification, the integration of the MLCA module, and the adoption of the EIoU loss function—enable the model to achieve a well-balanced trade-off among accuracy, efficiency, and robustness.

**Figure 8:**
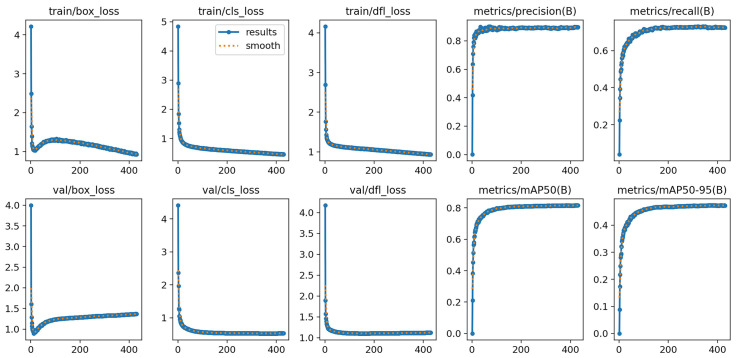
MG-YOLOv8 Training and Validation Performance Curves Chart.

To further validate the training stability and convergence behavior of the proposed MG-YOLOv8 model, the training and validation loss curves, as well as performance metrics such as precision, recall, and mAP, are illustrated in Figure 8. The curves show that both the classification and localization losses decrease steadily with the number of epochs, while the precision and mAP values rapidly converge to high levels, indicating strong training stability and efficient optimization.

### 4.4. Performance Evaluation of the Fatigue Detection Module

To evaluate the effectiveness of the proposed fatigue detection method, this study first validates the approach using video clips from the Mirror subset of the YawDD dataset. A total of 320 videos were categorized into three classes: normal state, talking state, and fatigue state. After excluding videos in which drivers wore sunglasses, 298 videos depicting volunteers of different ages and genders in a simulated driving environment were selected for further analysis. The results are presented in Table 8.

As shown in Table 8 and the graphical results, the proposed fatigue driving detection method demonstrates high accuracy and good real-time performance on the YAWDD dataset. It achieves an average recognition accuracy of 94.5% and maintains a stable frame rate of 30 FPS, meeting the requirements for practical deployment. While there are instances where normal states or conversational behaviors are misclassified as mild fatigue, the method consistently performs well in the fine-grained classification of fatigue states (e.g., mild, moderate, and severe fatigue). This demonstrates its strong detection capabilities and application potential.

On this basis, the performance of the proposed fatigue detection model was compared with previous studies on driver drowsiness detection reported in the literature, as summarized in Table 9. Since the YawDD dataset was used for experimental analysis in this work, only studies that conducted experiments on the same dataset were selected for comparison. In terms of classification accuracy, the proposed model outperforms existing methods in driver fatigue detection tasks.

To detect driver fatigue under complex driving conditions, reference [28] employed an improved YOLOv3-tiny network combined with facial feature triangles and feature vectors, achieving an accuracy of 94.32% on the YawDD dataset. In [35], the authors utilized MTCNN and Dlib for face detection and landmark localization, while LSTM was applied to extract temporal features, resulting in an accuracy of 88% on the same dataset. Reference [53] adopted a conventional convolutional neural network (CNN) to extract static facial features of drivers for classification, attaining an accuracy of 93.83%. In [32], a privacy-preserving federated transfer learning model (PFTL-DDD) was proposed for cross-domain fatigue detection, which achieved an accuracy of 86% on the YawDD dataset. Reference [31] used Dlib features in conjunction with a linear SVM classifier to identify fatigue based on blink duration analysis, reaching an accuracy of 92.5%. Finally, [39] introduced a two-stream spatiotemporal graph convolutional network (2s-STGCN) that integrates spatial structural and temporal dynamic features of facial landmarks, achieving a detection accuracy of 93.4% on the YawDD dataset.

**Figure 9:**
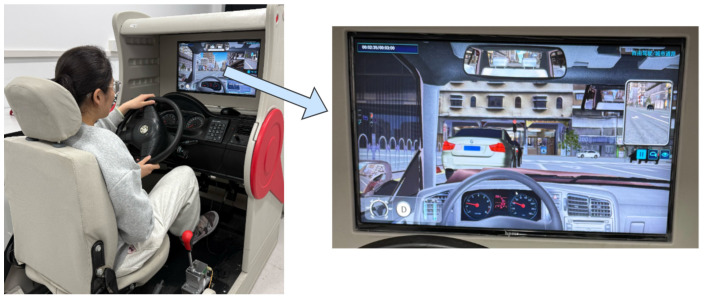
Driving simulator diagram.

After completing the validation on public datasets, we further conducted real-time fatigue detection experiments to evaluate the model’s performance under practical driving conditions. A total of 12 participants (aged 20–25 years, including 6 males and 6 females) were recruited to perform a series of predefined driving tasks in a simulated driving environment (see Figure 9), which included typical road scenarios such as urban streets and congested intersections. During the experiment, facial videos were continuously captured in real time using a Logitech C270 camera (Newark, CA, USA), and the model generated online outputs for fatigue-related behaviors such as blinking, yawning, and head nodding. To assess accuracy, the model’s detection results were compared frame by frame with manually annotated ground-truth behavioral data, and the average error rates for blinking, yawning, and nodding were computed accordingly. Table 10 presents the corresponding values of BF, PERCLOS, YF, and NF recorded for each participant during the experimental process.

Based on the results from the 12 test cases, the model outputs show a high level of consistency with the manually annotated ground-truth data, indicating strong overall detection accuracy. By comparing the model’s detection results with manual annotations across multiple trials, the average error rates for blink (BF), yawn (YF), and nod (NF) events were calculated as 1.5%, 5.2%, and 2.2%, respectively—representing an improvement over previous studies [55].

These findings demonstrate that the proposed model can stably and accurately capture key fatigue-related behaviors under real-time video conditions. The recognition accuracy for blinking and nodding remains particularly high, while yawning exhibits slightly greater variability due to differences in mouth movement amplitude and individual facial dynamics; however, the error remains within an acceptable range. The average error rate effectively reflects the model’s detection accuracy and robustness in real driving environments, confirming its adaptability and reliability under diverse conditions such as variable lighting, head poses, and partial occlusions.

**Figure 10:**
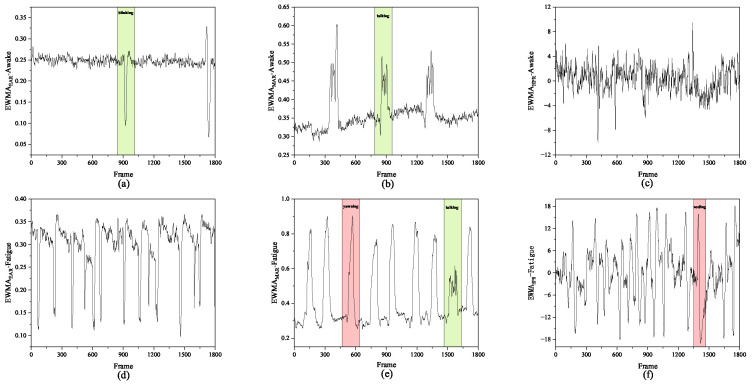
Curves of fatigue parameter changes in (**a**–**c**) awake state and (**d**–**f**) fatigue state in an extended monitoring period.

Figure 10 illustrates the temporal variations in the eye aspect ratio (EAR), mouth aspect ratio (MAR), and head posture angle (HPR) for different subjects during simulated driving experiments under both alert and fatigued conditions. As shown in Figure 10a, during the normal alert state, the EAR fluctuates slightly within a narrow range of 0.26–0.30, indicating stable eye-opening behavior. When the subject blinks or closes their eyes, the EAR curve exhibits a sharp drop, which quickly returns to the baseline once the eyes reopen—demonstrating the typical characteristics of a blink pattern. Figure 10b presents the temporal evolution of MAR: during speaking or mildly fatigued states, the MAR value oscillates slightly around 0.34, while during yawning, the MAR curve rises sharply with extended peak durations (exceeding 50 frames) and higher amplitudes compared to normal speech. This indicates that MAR effectively distinguishes normal speech from fatigue-induced yawning. Figure 10c shows the HPR variations in the alert state, where the curve remains stable, reflecting steady head posture without noticeable nodding behavior.

In contrast, Figure 10d–f depict the feature changes under fatigued conditions. As seen in Figure 10d, the baseline of the EAR curve decreases and its fluctuation frequency increases markedly, indicating more frequent and prolonged eye closures. In Figure 10e, the MAR curve displays more frequent and higher-amplitude peaks with extended durations, further confirming the increased occurrence of yawning during fatigue. Figure 10f shows that the HPR curve exhibits periodic and pronounced oscillations, corresponding to frequent head-nodding behavior and reduced postural stability—typical indicators of fatigue.

Overall, the three features—EAR, MAR, and HPR—exhibit distinct temporal patterns under fatigue: EAR decreases, MAR peaks intensify, and HPR fluctuations amplify. These dynamic variations align closely with physiological manifestations of fatigue such as eyelid drooping, frequent yawning, and head tilting. The results demonstrate that the proposed algorithm effectively captures and quantifies driver fatigue behaviors with high temporal sensitivity and physiological consistency.

## 5. Conclusions

This study proposes a multi-feature fusion fatigue detection method based on an improved YOLOv8 model. The system adopts a lightweight PFLD model to replace the conventional Dlib framework, achieving efficient and accurate facial landmark localization. By integrating head posture, eye, and mouth features, an innovative sliding window mechanism is designed to compute fatigue-related indicators in real time, including Percentage of Eye Closure (PERCLOS), Blink Frequency (BF), Yawn Frequency (YF), and Head Posture Ratio (HPR). The system subsequently employs a comprehensive fatigue evaluation algorithm to classify and promptly alert different levels of driver fatigue. Experimental results demonstrate that, on the YAWDD dataset, the proposed method achieves a detection accuracy of 94.5% with a stable inference speed exceeding 30 FPS. Moreover, the results of the driving simulator experiments indicate a low false detection rate, confirming that the proposed system effectively balances accuracy and real-time performance, making it highly suitable for deployment in resource-constrained embedded environments.

It is worth emphasizing that the configuration of feature weights plays a critical role in the overall detection performance. The current weight settings were determined through experimental validation and empirical tuning. However, future studies will conduct systematic investigations on larger and more diverse datasets, enabling fine-grained optimization of feature weights to further enhance model robustness and generalization capability. In addition, this work plans to expand the number of participants and experimental scenarios, introducing more comprehensive statistical performance metrics—such as Recall, and F1-Score—to achieve a more systematic and standardized evaluation of fatigue-state classification. These efforts will help verify the model’s effectiveness and stability under a broader range of real-world driving conditions. Furthermore, as the current system occasionally misclassifies speech-related mouth movements as yawning, future research will focus on dynamic mouth behavior modeling and the fusion of multimodal features (e.g., combining vocal and oral motion patterns) to improve recognition accuracy and adaptability in complex interactive environments.

## Figures and Tables

**Table 1:** Fatigue Detection Parameter Thresholds Table.

Parameter	EAR	MAR	HPR
EWMA Threshold	dynamic adjustment	0.75	10

**Table 2:** Evaluation Parameter Table.

Parameter	Description	Baseline	Max
F1	BF (times/min)	15	40
F2	PERCLOS (%)	5	50
F3	YF (times/min)	1	5
F4	NF (times/min)	3	10

**Table 3:** Ablation study on fusion weight configuration.

Group	$W_{1}$	$W_{2}$	$W_{3}$	$W_{4}$	Accuracy (%)
G1	0.25	0.25	0.25	0.25	91.13
G2	0.25	0.15	0.40	0.20	89.24
G3	0.15	0.30	0.33	0.22	93.17
G4	0.10	0.40	0.30	0.20	94.50
G5	0.12	0.45	0.28	0.15	93.10
G6	0.30	0.00	0.40	0.30	88.43
G7	0.00	0.45	0.55	0.00	91.81
G8	0.20	0.45	0.35	0.00	92.44
G9	1.00	0.00	0.00	0.00	75.65
G10	0.00	1.00	0.00	0.00	83.90
G11	0.00	0.00	1.00	0.00	77.62
G12	0.00	0.00	0.00	1.00	72.31

**Table 4:** Configuration and training environment.

Environment Parameter	Value
Operating system	Windows 11/64-bit
CPU	13th Gen Intel(R) Core(TM) i5-13490F (Intel, Chengdu, China)
RAM	16 GB
GPU	NVIDIA GeForce RTX 4070 Ti GPU (NVIDIA, Santa Clara, CA, USA)
Integrated development environment	PyCharm Community Edition 2022.1.1
Programming language	Python 3.8
Deep learning framework	PyTorch 1.13.1
Video capture camera	Logitech C270 HD Webcam (Logitech, Lausanne, Switzerland)

**Table 5:** Hyperparametric configuration.

Hyperparameter	Value
Learning rate	0.01
Image size	640 × 640
Momentum	0.937
Optimizer	SGD
Batch size	16
Epoch	500
Weight decay	0.0005

**Table 6:** Ablation experiments with the modules.

RepNCSPELAN4	MLCA	EIoU	P	mAP@0.5	Params (M)	GFLOPs
			0.889	0.830	11.13	28.6
✓			0.887	0.812	7.89	20.1
	✓		0.906	0.814	11.13	28.8
		✓	0.893	0.813	11.14	28.6
✓	✓		0.908	0.816	7.90	20.2
✓		✓	0.893	0.815	7.89	20.3
	✓	✓	0.909	0.817	11.15	28.9
✓	✓	✓	0.910	0.818	7.88	20.1

The tick symbol (✓) indicates that the corresponding module is included in the model.

**Table 7:** Performance comparison of different face detection methods on the WIDER FACE dataset.

Modules	P	mAP@0.5	Params (M)	GFLOPs
YOLOv5s	0.889	0.825	9.12	24.0
SSD	0.887	0.727	26.28	62.74
MTCNN [23]	0.880	0.757	3.90	4.20
Faster R-CNN	0.896	0.732	28.34	940.97
VJ [17]	0.701	0.294	0.003	0.05
YOLOv8s	0.889	0.830	11.13	28.6
YOLOv8s + MobileNetV3	0.852	0.706	2.50	6.8
MG-YOLOv8 (ours)	0.910	0.818	7.88	20.1

**Table 8:** Fatigue Detection Performance Evaluation Table.

Behavior Category	Number of Videos	Detected Fatigue Number			Accuracy (%)	Average Accuracy	Frame Rate
		Mild	Moder	Severe			
Normal	87	3	0	0	96.5	94.5	31.2 FPS
Conversation	86	6	1	0	91.8		
Fatigue	125	84	23	12	95.2		

**Table 9:** Proposed model’s comparison with other studies.

Reference	Methodology	Accuracy (%)
[28]	YOLOv3-tiny CNN + Face Feature Triangle + Face Feature Vector	94.32
[35]	MTCNN + DLIB + LSTM	88.00
[53]	Privacy-preserving federated transfer learning method (PFTL-DDD)	86.00
[32]	Dlib + Linear Support Vector Machine	92.50
[31]	2s-STGCN	93.40
[54]	CNN	93.83
Ours	MG-YOLOv8 + PFLD + Multi-feature Fusion	94.50

**Table 10:** Fatigue Detection Experimental Results Record Table.

Test Case	BF	Perclos	YF	NF	F	Detection Status	Actual Status
Case 1	19	0.08	2	1	0.10	Awake State	Awake State
Case 2	28	0.09	5	4	0.50	Moderate Fatigue	Moderate Fatigue
Case 3	17	0.14	4	7	0.28	Mild Fatigue	Mild Fatigue
Case 4	30	0.16	10	3	0.42	Moderate Fatigue	Moderate Fatigue
Case 5	8	0.30	2	0	0.19	Awake State	Awake State
Case 6	26	0.21	5	6	0.47	Mild Fatigue	Mild Fatigue
Case 7	32	0.48	3	8	0.75	Severe Fatigue	Severe Fatigue
Case 8	24	0.18	4	3	0.38	Mild Fatigue	Mild Fatigue
Case 9	22	0.29	0	4	0.22	Awake State	Awake State
Case 10	35	0.36	1	2	0.36	Mild Fatigue	Mild Fatigue
Case 11	14	0.05	2	3	0.075	Awake State	Awake State
Case 12	45	0.27	4	7	0.63	Moderate Fatigue	Moderate Fatigue

## Data Availability

The original contributions presented in this study are included in the article. Further inquiries can be directed to the corresponding author.
